# Molecular biomarkers screened by next-generation RNA sequencing for non-sentinel lymph node status prediction in breast cancer patients with metastatic sentinel lymph nodes

**DOI:** 10.1186/s12957-015-0642-2

**Published:** 2015-08-28

**Authors:** Feng Liang, Hongzhu Qu, Qiang Lin, Yadong Yang, Xiuyan Ruan, Bo Zhang, Yi Liu, Chengze Yu, Hongyan Zhang, Xiangdong Fang, Xiaopeng Hao

**Affiliations:** Affiliated Hospital of Academy of Military Medical Sciences, 8 Dongdajie, Fengtai District, Beijing, 100071 China; CAS Key Laboratory of Genome Sciences and Information, Beijing Institute of Genomics, Chinese Academy of Sciences, 1-104 Beichen West Road, Chaoyang District, Beijing, 100101 China; General Hospital of Beijing Military Area, 5 Nanmencang, Dongcheng District, Beijing, 100700 China

**Keywords:** Breast cancer, Sentinel lymph node (SLN), Non-sentinel lymph node (NSLN), Axillary lymph node dissection (ALND), RNA sequencing

## Abstract

**Background:**

Non-sentinel lymph node (NSLN) status prediction with molecular biomarkers may make some sentinel lymph node (SLN) positive breast cancer patients avoid the axillary lymph node dissection, but the available markers remain limited.

**Methods:**

SLN positive patients with and without NSLN invasion were selected, and genes differentially expressed or fused in SLN metastasis were screened by next-generation RNA sequencing.

**Results:**

Six candidates (all ER/PR+, HER2−, Ki-67 <20 %) with metastatic SLNs selected from 305 patients were equally categorized as NSLN negative and positive. We identified 103 specifically expressed genes in the NSLN negative group and 47 in the NSLN positive group. Among them, *FABP1* (negative group) and *CYP2A13* (positive group) were the only 2 protein-encoding genes with expression levels in the 8th to 10th deciles. Using a false discovery rate threshold of <0.05, 62 up-regulated genes and 98 down-regulated genes were discovered in the NSLN positive group. Furthermore, 10 gene fusions were identified in this group with the most frequently fused gene being *IGLL5*.

**Conclusions:**

The biomarkers screened in present study may broaden our understanding of the mechanisms of breast cancer metastasis to the lymph nodes and contribute to the axillary surgery selection for SLN positive patients.

**Electronic supplementary material:**

The online version of this article (doi:10.1186/s12957-015-0642-2) contains supplementary material, which is available to authorized users.

## Background

Axillary lymph node dissection (ALND) was introduced as a standard surgical procedure for breast cancer in the 1800s and played a significant role in patients’ staging, prognosis assessment, regional disease control, and treatment direction [1, 2]. However, with the aid of new screening methods, more early stage patients with no invasion of axillary lymph nodes (ALN) have been able to be identified in recent years. For these patients, instead of reducing the incidence of recurrence or improving survival, ALND was found to be associated with increased risk of adverse effects such as lymphedema, limited mobility, neuropathic pain, numbness, and sensory loss [3–5]. To solve this dilemma, sentinel lymph node biopsy (SLNB), a less invasive surgery with equivalent clinical value, was developed and has readily become a routine surgery in early breast cancer patients [6, 7].

As the first site of tumor cell infiltration via lymphatic vessels, sentinel lymph nodes (SLN) with no detectable metastasis are seen as safety indicator, thus, making ALND unnecessary. On the other hand, if SLN is positive for metastasis, ALND is still recommended to clarify the status of the remaining non-sentinel lymph nodes (NSLNs) in the axilla [8, 9]. Nevertheless, it was reported that 40–70 % of SLN positive patients were actually free of metastasis in their NSLNs [10, 11]. In order to avoid the over-treatment suffering brought about by ALND, it has become imperative for breast cancer surgeons to find effective methods that can distinguish SLN positive patients with low probability of NSLN invasion from those with high probability of NSLN invasion.

Among these methods, predictive models based on retrospective analysis of patients’ clinical characteristics (e.g., age, histological type, tumor size, lymphovascular invasion, and hormone receptor status), such as the nomogram of Memorial Sloan-Kettering Cancer Center [12] and the scoring systems of MD Anderson [13], Tenon [14], Cambridge [15], and Stanford [16], were the most frequently mentioned ones. However, the routine clinical practice and patient characteristics varied among different hospitals, thereby, greatly influencing the accuracy, consistency, and repeatability of these models and hampering their extensive application. On the other hand, it was hypothesize that tumor with specific gene expression or fusion may have more invasive behavior and thus possess with higher possibility of metastasis in lymph node. Therefore, some scientists were dedicated to search for biomarkers that can predict NSLN status [17–23], but until recently, the available choices remained limited and their practical value still needed additional verification.

In present study, next-generation RNA sequencing (RNA-Seq) was utilized to compare gene expression level differences for breast cancer metastasized to the SLN between patients with and without NSLN invasion. To our knowledge, it is the first time that NSLN prediction markers were screened according to gene expression profiling of the SLN metastasis. Although further validation is required in the future, these markers could broaden our understanding of the mechanisms of breast cancer metastasis to the lymph nodes and might provide assistance in decision making when choosing appropriate surgery strategies for SLN positive breast cancer patients.

## Methods

### Patients

Treatment-naive breast cancer patients who received SLNB at our hospital were selected for the present study. Among them, patients with metastatic SLN were divided into NSLN positive and negative groups based on their ALND results. For traditional clinical indexes such as age, tumor size, histological type, and numbers of metastatic SLN and ALN, as well as estrogen receptor (ER), progesterone receptor (PR), HER2, Ki-67 status, and patients with greatly varying characteristics, were excluded from each group. For the remaining patients in the two refined groups, 10 slices (4–5 μm) of paraffin embedded SLN samples were collected for subsequent analysis. To participate in the study, all patients signed an informed consent form that was approved by the ethics and scientific committees at the affiliated hospital of Academy of Military Medical Sciences.

### RNA extraction, library preparation, and sequencing

Using the delineation line drawn by the pathologist on the reverse side of each slice as a guide, the metastatic tumor in the SLN was scraped into a 1.5 ml RNase-free tube and sent for RNA extraction using the RNeasy FFPE kit (Qiagen, Germany) according to the manufacturer’s instructions. The obtained total RNA was measured using a NanoDrop 2000 (Thermo Scientific, USA) and stored at −80 °C until used. Libraries of mRNA derived from total RNA were constructed using the Illumina ®TruSeq™ RNA Sample Preparation Kit (USA) according to the manufacturer’s instructions. The concentration and size distribution of the libraries were determined using an Agilent 2100 Bioanalyzer (USA). The libraries were then sequenced using an Illumina Hiseq 2000 Genome Analyzer platform in paired-end 100-bp mode.

### Data analysis

Sequenced reads were processed and aligned to the UCSC reference human genome (build hg19) using the Tophat software [24] default setting and were then fed to Cufflinks software [25] to assemble transcripts and estimate their abundances. To calculate gene expression levels, read counts were normalized to the number of fragments per kilobase of transcript per million mapped reads (FPKM) according to the gene length and total mapped reads. The unsupervised hierarchical clustering of gene expression levels from the selected samples and the final dendrogram visualization were performed using the R programming package. The Cuffcompare program was used to track the expression levels of each transcript within samples and to produce a combined gene expression file. This file was then run through the Cuffdiff program to test for differences in gene expression in breast cancer metastasized to SLN between patients with and without NSLN invasion. First, specifically expressed genes were identified as being expressed in the NSLN negative or positive group exclusively. They were divided into lowly (1st–3rd decile), moderately (4th–7th decile), and highly (8th–10th decile) expressed genes according to their expression levels. The non-specific genes were used to further filter down- and up-regulated genes with a false discovery rate (FDR) <0.05. Gene Ontology function classifications of regulated genes were assigned using DAVID (*p* ≤ 0.001) [26]. Fusion genes were searched using Tophat with “--fusion-search” specified during the process of read alignment [27]. A “supporting” read must map to both sides of a fusion by at least 13 bases. For intra-chromosomal fusions, the distance between the fusion points must be at least 100 kb. Reads or pairs that map to more than two places were ignored. The final fusion genes with ≥5 supporting reads and pairs were identified in the end.

## Results

### Patient characteristics

Sixty-nine SLN positive breast cancer patients were chosen from 305 patients who received SLNB between November 2010 and April 2013 at our hospital. Among them, 32 patients were NSLN positive and the other 37 patients were NSLN negative. Based on their clinical indexes, 3 patients were selected from each group for subsequent research. The characteristics of the 6 patients are listed in Table 1. Their backgrounds were generally the same: all were moderately differentiated invasive ductal carcinoma (IDC), with positive ER/PR, and negative HER2. For Ki-67, the requirements had to be broadened to ≤20 %, since there were insufficient patients in the NSLN negative group when the recommended cut-off point of 14 % was used [28].

**Table 1:** The characteristics of the selected patients

	NSLN negative			NSLN positive		
Patient ID	84816	94948	67161	76948	86923	94812
Tumor type	IDC	IDC	IDC	IDC	IDC	IDC
Tumor grade	MD	MD	MD	MD	MD	MD
Tumor size (cm)	1.5	1	3	3	2	2.5
SLN (P/T)	1/2	1/4	1/1	2/2	1/1	1/2
ALN (P/T)	0/18	0/16	0/18	1/21	2/21	1/28
ER	3+, >75 %	3+, >75 %	2+, 50–70 %	2+, 50–75 %	3+, >75 %	3+, >75 %
PR	3+, >75 %	1+, ~15 %	1+, 10–30 %	1+, 25–50 %	2+, 50–60 %	3+, >75 %
HER2	Negative	Negative	Negative	Negative	Negative	Negative
Ki-67	3–5 %	20 %	15–20 %	10 %	10 %	5–10 %

*IDC* invasive ductal carcinoma, *MD* moderate differentiated, *P* positive, *T* total

### RNA extraction, library preparation, and sequencing

**Fig. 1:**
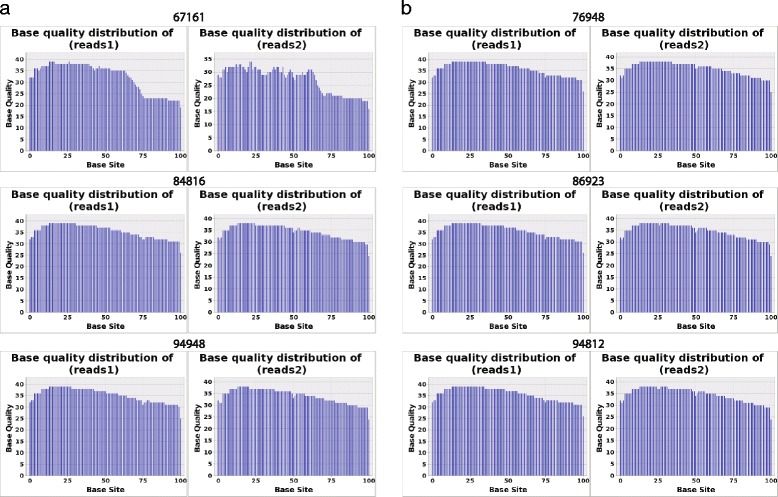
Distribution of sequencing base quality. **a** Base quality of paired-end reads for NSLN negative group. **b** Base quality of paired-end reads for NSLN positive group. The base quality of sequencing reads was greater than 25 in five samples except for 67167 whose base quality was slightly lower (≥20)

As showed in Additional file 1, the extracted RNA concentrations for each sample were all >100 ng/μl and their OD260/OD280 ratio ranged from 1.78 to 2.03, which ensured that the samples could be used for downstream experiments. We successfully generated cDNA libraries of 350–500 bp and obtained 18–27 million (range 18,549,392–27,137,861; mean 22,775,012) high-quality sequencing reads with a sequencing quality of >25 for each base in five samples and >20 in sample 67161 (Fig. 1). The raw sequence data has been deposited in a public repository (Gene Expression Omnibus (GEO)) with the access number GSE64850. After filtering the repetitive or very low complexity reads (0.16 % of the sequenced reads on average), we mapped an average of 52.27 % (range 19.57–67.22 %) of the reads to the human genome (UCSC version hg19) (Additional file 2).

### Variation in gene expression

**Fig. 2:**
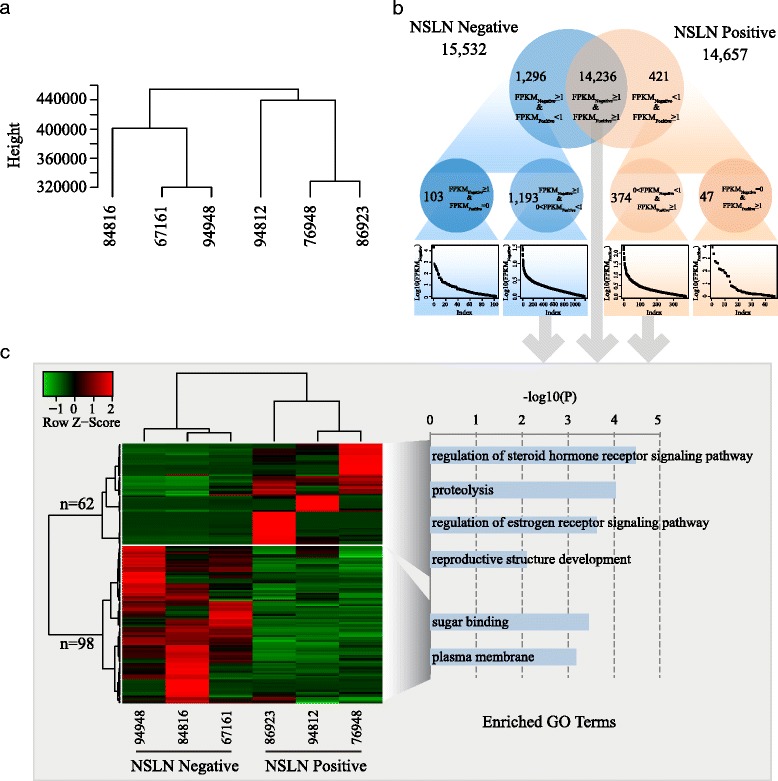
Gene expression variations between NSLN negative and positive groups. **a** Clustering of gene expression levels in the six samples categorized them in a manner consistent with their clinical traits. **b** Venn diagram of genes with FPKM ≥ 1 in the two groups. There are more NSLN negative group-specific genes than NSLN positive group-specific genes. **c** Heatmap of 62 up-regulated genes and 98 down-regulated genes in the NSLN positive group and their enriched GO terms

We used FPKM values to measure the gene expression level, which can compensate for biases between samples. Considering that the expression levels of a gene may not be accurately detected by RNA-Seq when its FPKM value is less than 1, only those genes with FPKM values ≥1 were considered for subsequent analysis. The unsupervised hierarchical clustering of gene expression levels clearly categorized the six patients into the NSLN negative or NSLN positive group in a manner consistent with their clinical traits (Fig. 2a). Furthermore, NSLN negative samples displayed highly similar gene expression profiles (Additional file 3) as supported by the Pearson correlation of gene expression values: 0.8, 0.72, and 0.83 for 67167 vs. 84816, 67167 vs. 94948, and 84816 vs. 94948, respectively. However, NSLN positive samples showed a much greater heterogeneity in gene expression than NSLN negative ones because of the more diverse sample 76948 (Pearson correlation coefficients were 0.63 for 76948 vs. 86923 and 0.44 for 76948 vs. 94812, respectively).

In order to identify the important and specifically expressed genes, we first classified genes with FPKM ≥ 1 in each group into lowly, moderately, and highly expressed genes (Additional file 4). Then we explored genes exclusively expressed in each group (Fig. 2b) and examined their expression category (Additional file 5). In the NSLN negative group, 103 genes were specifically expressed and 12 of them with highly expression (11 were RNA genes and 1 was a protein-coding gene, *FABP1*). In contrast, 47 genes were specifically expressed in the NSLN positive group, 13 of which with highly expression, including 12 non-coding RNA genes (4 Micro RNAs, 8 small nucleolar RNAs) and 1 protein-coding gene, *CYP2A13* (see Table 2).

**Table 2:** Specifically and highly expressed genes in the NSLN negative and positive groups

Gene	FPKM	Description	Category
13 NSLN-positive-specific			
MIR3936	2210.6	MicroRNA 3936	RNA gene
MIR223	7256.4	MicroRNA 223	RNA gene
SNORA3	569	Small nucleolar RNA, H/ACA box 3	RNA gene
SNORA18	433.8	Small nucleolar RNA, H/ACA box 18	RNA gene
MIR941-3	409.7	MicroRNA 941-3	RNA gene
SNORA7B	143.2	Small nucleolar RNA, H/ACA box 7B	RNA gene
SNORA13	131.6	Small nucleolar RNA, H/ACA box 13	RNA gene
SNORA2A	113.8	Small nucleolar RNA, H/ACA box 2A	RNA gene
SCARNA11	112	Small nucleolar RNA, H/ACA box 11	RNA gene
MIR3907	76	MicroRNA 3907	RNA gene
SNORA84	66.7	Small nucleolar RNA, H/ACA box 84	RNA gene
CYP2A13	39.6	Cytochrome P450, family 2, subfamily A, polypeptide 13	Protein-coding
SNORA62	38.9	Small nucleolar RNA, H/ACA box 62	RNA gene
12 NSLN-negative-specific			
SNORD89	20306.9	Small nucleolar RNA, C/D box 89	RNA gene
MIR499A	683.4	MicroRNA 499a	RNA gene
SNORA51	452.6	Small nucleolar RNA, H/ACA box 51	RNA gene
SNORA40	419.3	Small nucleolar RNA, H/ACA box 40	RNA gene
SNORA46	281.1	Small nucleolar RNA, H/ACA box 46	RNA gene
SCARNA3	206.1	Small Cajal body-specific RNA 3	RNA gene
SNORA27	181.4	Small nucleolar RNA, H/ACA box 27	RNA gene
SNORA32	99.9	Small nucleolar RNA, H/ACA box 32	RNA gene
FABP1	84.2	Fatty acid binding protein 1, liver	Protein-coding
MIR941-2	43.3	MicroRNA 941-2	RNA gene
MIR941-4	43.3	MicroRNA 941-4	RNA gene
SNORA1	43	Small nucleolar RNA, H/ACA box 1	RNA gene

From the remaining 15,803 non-specific genes (Fig. 2b, 14,236 + 1193 + 374), we further identified 62 up-regulated and 98 down-regulated genes in the NSLN positive group using the Cuffdiff software with a threshold of FDR < 0.05 (Fig. 2c and Additional file 6). This was consistent with the overall expression profiling of all genes. Among the 160 genes, the top 10 of differentially expressed genes were listed in Table 3. Genes involved in reproductive structure development (*p* = 0.008), proteolysis (*p* = 9.19e-5), regulation of steroid hormone receptor signaling pathway (*p* = 3.4e-5), and regulation of estrogen receptor signaling pathway (*p* = 2.4e-4) were enriched in the up-regulated gene group, including four kallikrein-related peptidase (KLK) subfamily members (*KLK10*, *KLK11*, *KLK12*, and *KLK13*), whereas genes involved in sugar binding (*p* = 3.5e-4), the plasma membrane (*p* = 6.6e-4) (Fig. 2c), and the B cell receptor antigen signaling pathway (FDR = 3.63e-10) were enriched in the down-regulated gene group (Additional file 7).

**Table 3:** Top ten of differentially expressed genes in the NSLN positive group

Gene	NSLN negative	NSLN positive	Log2(FC)	FDR	Description
Up-regulated genes					
KLK11	0.93	185.48	7.64	0.00015	Kallikrein-related peptidase 11
SCGB3A1	8.06	1231.39	7.26	4.89E-05	Secretoglobin, family 3A, member 1
CLEC3A	0.98	143.59	7.19	2.96E-06	C-type lectin domain family 3, member A
CYP2A6	0.45	63.31	7.12	0.00029	Cytochrome P450, family 2, subfamily A, polypeptide 6
KLK10	0.17	19.11	6.80	0.0034	Kallikrein-related peptidase 10
KLK12	0.89	94.90	6.73	0.0011	Kallikrein-related peptidase 12
KLK13	0.57	49.98	6.45	7.42E-05	Kallikrein-related peptidase 13
CYP2A7	0.33	26.37	6.32	0.0052	Cytochrome P450, family 2, subfamily A, polypeptide 7
OBP2B	0.43	33.39	6.29	0.0391	Odorant binding protein 2B
KCNC2	0.32	21.60	6.09	0.0040	Potassium voltage-gated channel, Shaw-related subfamily, member 2
Down-regulated genes					
KRT20	17.19	0.07	−7.95	0.00019	Keratin 20
KRT4	6.42	0.05	−7.14	0.048	Keratin 4
VPREB1	25.93	0.19	−7.10	0.012	Pre-B lymphocyte 1
RBP2	31.24	0.58	−5.75	0.0014	Retinol binding protein 2, cellular
ALDOB	4.64	0.09	−5.66	0.011	Aldolase B, fructose-bisphosphate
BIRC7	7.80	0.16	−5.57	0.017	Baculoviral IAP repeat containing 7
FIBCD1	7.75	0.19	−5.38	0.00091	Fibrinogen C domain containing 1
MUC13	8.85	0.23	−5.24	0.0022	Mucin 13, cell surface associated
FCAMR	50.50	1.38	−5.19	4.97E-12	Fc receptor, IgA, IgM, high affinity
DHRS2	19.04	0.55	−5.10	0.0085	Dehydrogenase/reductase (SDR family) member 2

*FC* fold change of (NLSN positive/negative)

### Fusion gene

**Fig. 3:**
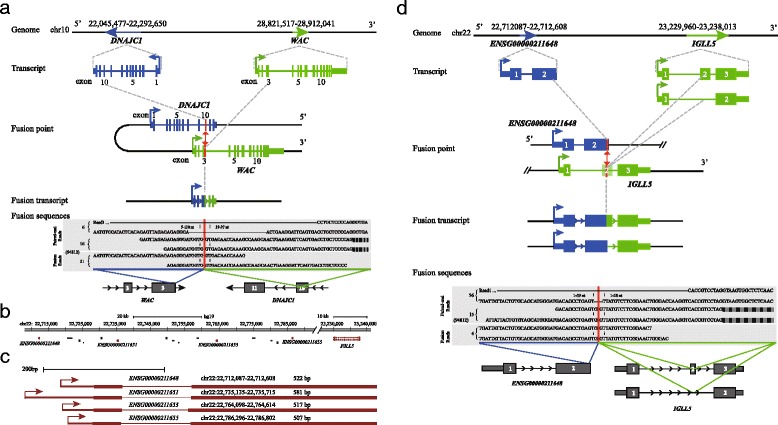
Examples of fusion genes in NSLN positive samples. **a** The *WAC*-*DNAJC1* fusion identified only in 94812 was revealed by 21 fusion-spanning reads, 16 pair-end reads (with one read spanning the fusion point), and 6 pair-end reads. This fusion was between one-third of exon 3 of *WAC* (*blue*) and the whole of exon 10 of *DNAJC1* (*green*). The break point was shown in a *red vertical line*. The numbers of reliable pair-end and fusion-spanning reads in each sample are indicated to the right of each read. The sample ID is showed in parentheses. **b** Schematic diagram of genomic locations of *IGLL5* and four ensemble non-coding genes fused with *IGLL5*. Five genes with the fusion are shown in *red*. **c** The similar structure for four IGLV1 variables fused with IGLL5. These four genes were aligned to the right and the name, position, and length were shown above each gene. **d** One example of *IGLL5* fusion with a variable of *IGLV1*, *ENSG00000211648* that was only identified in 94812. There are two transcripts of *IGLL5* gene and two fused transcripts with *ENSG00000211648* correspondingly

A total of 10 different gene fusions were identified in the NSLN positive group, including 7 fusions taking place only in 94812, 2 fusions happening only in 76948, and 1 fusion occurring only in 86923 (Table 4). The intra-chromosome gene fusion *WAC*-*DNAJC1* that occurred only in 94812 was located between a part of exon 3 of *WAC* and the whole of exon 10 of *DNAJC1* (Fig. 3a). The inter-chromosome gene fusion *CACNG4*-*RANBP3* that occurred in 86923 was located in the intron sequence between exons 1 and 2 of both *CACNG4* and *RANBP3* (Additional file 8). The *PDE3A*-*SLCO5A1* gene fusion in 76948 was also localized between the introns of both genes (Additional file 8). The remaining 7 gene fusions were formed through fusion of one formal gene and an ensemble gene (Table 4). Interestingly, the most frequently fused gene was *IGLL5* (immunoglobulin lambda-like polypeptide 5) that fused with four variants of the *IGLV1* (partial mRNA for immunoglobulin lambda light chain) gene. These four variants were located in a 75 kb region about 445 to 520 kb upstream of *IGLL5* (Fig. 3b) and exhibit similar gene structure (Fig. 3c). The fusion point for *IGLL5* was in the intron region of one transcript and the exon 2 region of the other transcript, while fusion points for the *IGLV1* variants were located in the exon 2 regions (Fig. 3d).

**Table 4:** Fusion genes identified in NSLN positive samples

Gene	Chrom	Sample ID	Reads #
*WAC*-*DNAJC1*	chr10-chr10 fr	94812	43
*ENSG00000211648*(*IGLV1-47*)-*IGLL5*	chr22-chr22 ff	94812	55
*ENSG00000211651*(*IGLV1-44*)-*IGLL5*	chr22-chr22 ff	94812	110
*ENSG00000211653*(*IGLV1-40*)-*IGLL5*	chr22-chr22 ff	94812	65
*ENSG00000211655*(*IGLV1-36*)-*IGLL5*	chr22-chr22 ff	94812	70
*ENSG00000230613*(*HM13-AS1*)-*HM13*	chr20-chr20 rf	94812	20
*SSB*-*ENSG00000236852*(*RP11-3D23.1*)	chr2-chrX rr	94812	28
*PDE3A*-*SLCO5A1*	chr12-chr8 rr	76948	9
*ENSG00000226958*(*CTD-2328D6.1*)-*HFM1*	chrX-chr1 rr	76948	50
*CACNG4*-*RANBP3*	chr17-chr19 ff	86923	24

fr stands for fusion occurring between forward strand in the first chromosome with reverse strand in the second chromosome. ff stands for fusion occurring between forward strands in both chromosomes. rr stands for fusion occurring between reverse strands in both chromosomes

## Discussion

Because of growing evidence for its benefits and its minor side effects in patients, SLNB has readily replaced ALND and has become the routine procedure for surgical axillary staging in early breast cancer patients [6, 7]. For SLN negative patients, it is now widely accepted that ALND can be omitted [8, 9]. Because 40–70 % of SLN positive patients were reported to be free of metastasis in their NSLN, ALND in these patients remains controversial [10, 11]. In order to avoid the physical discomfort and potential complications associated with ALND in these patients, an effective method to predict the status of NSLN has become the urgent demand for breast surgeon. In contrast to the existing predictive models that are based on retrospective analysis of patients’ clinical characteristics [12–16], molecular tests may hold significant promise because they are more objective, more standardized, and easier to popularize [17–23]. Unfortunately, currently available markers remain limited and their practical value still needs additional verification.

Recently, the utilization of RNA-Seq in breast cancer has illustrated its power in revealing the variation landscape of the breast transcriptome and in finding regulatory interactions among cancer-related molecules [29, 30]. As a powerful next-generation sequencing technology, RNA-Seq can profile a full set of transcripts including mRNAs, small RNAs, and other non-coding RNAs qualitatively and quantitatively, providing a snapshot of gene expression patterns and regulatory elements in a cell, tissue, or organism. Compared with microarrays, RNA-Seq possesses the advantages of being high-throughput, cost effective, and of having superior accuracy. In addition, without relying on prior sequence information, RNA-Seq can profile gene expression based on the entire transcript (not a few segments). It can also identify novel isoforms and exons, allele-specific expression, mutations, and fusion transcripts [31]. These advantages make it ideal for studying complex diseases, particularly cancer. Despite its growing application in breast cancer, to the best of our knowledge, the present study is the first one using RNA sequencing to screen for potential markers predicting NSLN status in patients with metastatic SLN.

The major function and most distinctive feature of RNA-Seq is measuring gene expression variance, which captures the genetic differences among patients. The most interesting observation in our study is that four KLK subfamily members (*KLK10*, *KLK11*, *KLK12*, and *KLK13*) were up-regulated in the NSLN positive group, suggesting their potential role in lymph node metastasis. The KLK gene family includes 15 highly conserved secreted serine proteases with similar structural characteristics, whose dysregulation was reported to be closely associated with endocrine-related cancer, such as prostate, breast, and ovarian cancers [32]. Although previous studies have demonstrated the crucial role of *KLK10* and *KLK11* in breast cancer patients’ relapse, disease progression and shorter survival rates [32, 33], a potential role for the KLK gene family in lymph node metastasis was first proposed in the present study. More studies are required to further confirm these results.

On the other hand, for the down-regulated genes in the NSLN positive group, B cell antigen receptor (BCR) signaling pathway, including some B cell surface molecules (CD22, CD72, Igα, Igβ, CD19, and CD21) and a few downstream regulated genes (*SYK*, *LYN*, *BTK*, and *PTPN6*), may be worthy of further attention. It is known that the BCR signal pathway is vital for the development and survival of B lymphocytes and that defective BCR signaling can result not only in impaired B cell development and immunodeficiency but also in a predisposition to autoimmunity [34]. Although the BCR signaling pathway was previously reported to play significant roles in chronic lymphocytic leukemia [35], this is the first time that it is linked with NSLN metastasis in breast cancer.

In contrast to the down- and up- regulated genes, the presence of specifically expressed genes and fusion genes may be more useful to the breast cancer surgeon, because they are relatively easier to analyze and their detection could be carried out during surgery, thereby, facilitating the implementation of appropriate surgery strategies for breast cancer patients in a timelier manner. For specifically expressed genes, two protein-coding genes, *FABP1* and *CYP2A13* which were expressed in the NSLN negative and positive groups, respectively, were worthy of further investigation. *FABP1* was reported to correlate with non-alcoholic fatty liver disease [36], and *CYP2A13* was found to be involved in the development and progression of lung adenocarcinoma [37]; however, neither of them was previously associated with NSLN metastasis. For fusion genes, the most frequently seen in the NSLN positive group was *IGLL5*, which was identified as one of the best predictors for relapse-free survival with >85 % accuracy in breast cancer patients [38]. This observation suggests that those rearrangements occurring in *IGLL5* might be linked to the process of metastasis.

As a well-known biomarker for cell proliferation, Ki-67 plays a significant role in prognosis prediction [39] and has been routinely used in the subtyping of breast cancer [28]. However, we could not screen enough patients in the NSLN negative group using the recommended cut-off of 14 % [28]. Taking into consideration that such a cut-off was arbitrarily determined and still needed further confirmation, we broadened the requirement to 20 %. Even so, only six patients were finally screened, which may inevitably influence the strength of our results. Therefore, further verification in subsequent studies is required. Moreover, other subtypes of breast cancer (such as HER2 positive) were not evaluated in the present study and may need additional investigation, since their intrinsic metastatic mechanism may be completely different.

Lastly, we should note that predicting NSLN status with molecular biomarkers is based on the hypothesis that tumor with specific gene expression or fusion may have more invasive behavior and thus possess with higher possibility of metastasis in lymph node. However, specific gene expression or fusion in SLN does not necessarily mean the invasion of NSLN and that merely represents some kind of possibility. Therefore, as regards for the practical value of the biomarkers that screened in present study, additional verification should be warranted in the future.

## Conclusions

In summary, this is the first time that molecular markers for NSLN status prediction in SLN positive breast cancer patients were identified using transcriptome sequencing. These markers could broaden our understanding of the mechanisms of breast cancer metastasis to the lymph nodes. More importantly, the specifically expressed genes (*FABP1* and *CYP2A13*) and the fused gene (*IGLL5*) identified in our study may be integrated into an intro-operative diagnostic method, which could facilitate the implementation of appropriate surgery strategies in a timely manner.

## Additional files

Additional file 1:
**The results of RNA extraction.** A table listing the quality of RNA extraction. Additional file 2:
**Statistic information of sequencing reads in selected patients.** A table showing how the sequencing reads mapped. Additional file 3:
**Scatter plots of gene expression values between samples.** A figure illustrating how similar of genes expressed among 6 samples. Panels a, b, and c show comparisons among 67161, 94948, and 84816 from the NSLN negative group. These three samples display very similar gene expression levels based on Pearson correlations. Panels d, e, and f show comparisons among 76948, 86923, and 94812 from the NSLN positive group. Gene expression levels in 76948 are quite dissimilar compared with those in 86923 and 94812 based on their Pearson correlations. Additional file 4:
**Classification of genes based on the expression levels.** A table showing how the gene expression levels were classified. Additional file 5:
**Specifically expressed genes in the NSLN negative and –positive groups.** A table showing the FPKM values of specifically expressed genes in two groups. Additional file 6:
**Expression levels of 98** **down-regulated** **genes and 62** **up-regulated** **genes in the NSLN positive group.** A table showing the expression values of regulated genes. Additional file 7:
**A KEGG pathway figure for the enriched pathway B cell receptor signaling pathway in** **down-regulated** **genes.** A figure illustrating the pathway of down-regulated genes enriched. The genes colored with green were down-regulated in NSLN positive group. Additional file 8:
**The schematic diagram of other fused genes.** A figure showing how the rest of gene fusions occurred. The two genes that fused together were shown in blue and green. There fused point was shown in a vertical red line.

## Abbreviations

ALN: axillary lymph nodes
ALND: axillary lymph node dissection
BCR: B cell antigen receptor
ER: estrogen receptor
FDR: false discovery rate
FPKM: fragments per kilobase of transcript per million mapped reads
IDC: invasive ductal carcinoma
KLK: kallikrein-related peptidase
NSLN: non-sentinel lymph node
PR: progesterone receptor
RNA-Seq: RNA sequencing
SLN: sentinel lymph node
SLNB: sentinel lymph node biopsy
