# Supplementary material for: Molecular biomarkers screened by next-generation RNA sequencing for non-sentinel lymph node status prediction in breast cancer patients with metastatic sentinel lymph nodes
Source: World J Surg Oncol. 2015 Aug 28;13:258. doi: 10.1186/s12957-015-0642-2 (PMC4551378; doi:10.1186/s12957-015-0642-2)
Supplement: Additional file 1: — The results of RNA extraction. A table listing the quality of RNA extraction. [file 12957_2015_642_MOESM1_ESM.doc]

Additional file 1 The results of RNA extraction

| Patient ID | Concentration (ng/μl) | OD260/OD280 |
| --- | --- | --- |
| 84816 | 122.0 | 1.93 |
| 94948 | 307.2 | 2.03 |
| 67161 | 131.3 | 1.91 |
| 76948 | 127.5 | 1.78 |
| 86923 | 144.2 | 1.93 |
| 94812 | 150.6 | 1.99 |
