# Supplementary material for: Molecular biomarkers screened by next-generation RNA sequencing for non-sentinel lymph node status prediction in breast cancer patients with metastatic sentinel lymph nodes
Source: World J Surg Oncol. 2015 Aug 28;13:258. doi: 10.1186/s12957-015-0642-2 (PMC4551378; doi:10.1186/s12957-015-0642-2)
Supplement: Additional file 2: — Statistic information of sequencing reads in selected patients. A table showing how the sequencing reads mapped. [file 12957_2015_642_MOESM2_ESM.doc]

Additional file 2 Statistic information of sequencing reads in selected patients

| Sample | | | Total Reads | Filtered out | Processed reads | Mapped reads | Mapping (%) |
| --- | --- | --- | --- | --- | --- | --- | --- |
| NSLN  Negative | 67161 | Left | 18,549,392 | 38,490 | 18,510,902 | 3,752,195 | 20.27 |
|  |  | Right | 18,549,392 | 53,498 | 18,495,894 | 3,489,519 | 18.87 |
|  | 84816 | Left | 24,090,720 | 107,184 | 23,983,536 | 11,564,145 | 48.22 |
|  |  | Right | 24,090,720 | 107,184 | 23,963,438 | 11,113,294 | 46.38 |
|  | 94948 | Left | 21,737,633 | 2,233 | 21,735,400 | 12,176,454 | 56.02 |
|  |  | Right | 21,737,633 | 19,166 | 21,718,467 | 11,610,874 | 53.46 |
| NSLN  Positive | 76948 | Left | 23,236,958 | 8,292 | 23,228,666 | 14,252,662 | 61.36 |
|  |  | Right | 23,236,958 | 26,572 | 23,210,386 | 13,827,551 | 59.57 |
|  | 86923 | Left | 21,897,505 | 4,315 | 21,893,190 | 14,966,962 | 68.36 |
|  |  | Right | 21,897,505 | 21,589 | 21,875,916 | 14,452,401 | 66.07 |
|  | 94812 | Left | 27,137,861 | 1,243 | 27,136,618 | 17,820,515 | 65.67 |
|  |  | Right | 27,137,861 | 22,586 | 27,115,275 | 17,063,604 | 62.93 |
