# Supplementary figures and images for: Molecular biomarkers screened by next-generation RNA sequencing for non-sentinel lymph node status prediction in breast cancer patients with metastatic sentinel lymph nodes
Source: World J Surg Oncol. 2015 Aug 28;13:258. doi: 10.1186/s12957-015-0642-2 (PMC4551378; doi:10.1186/s12957-015-0642-2)

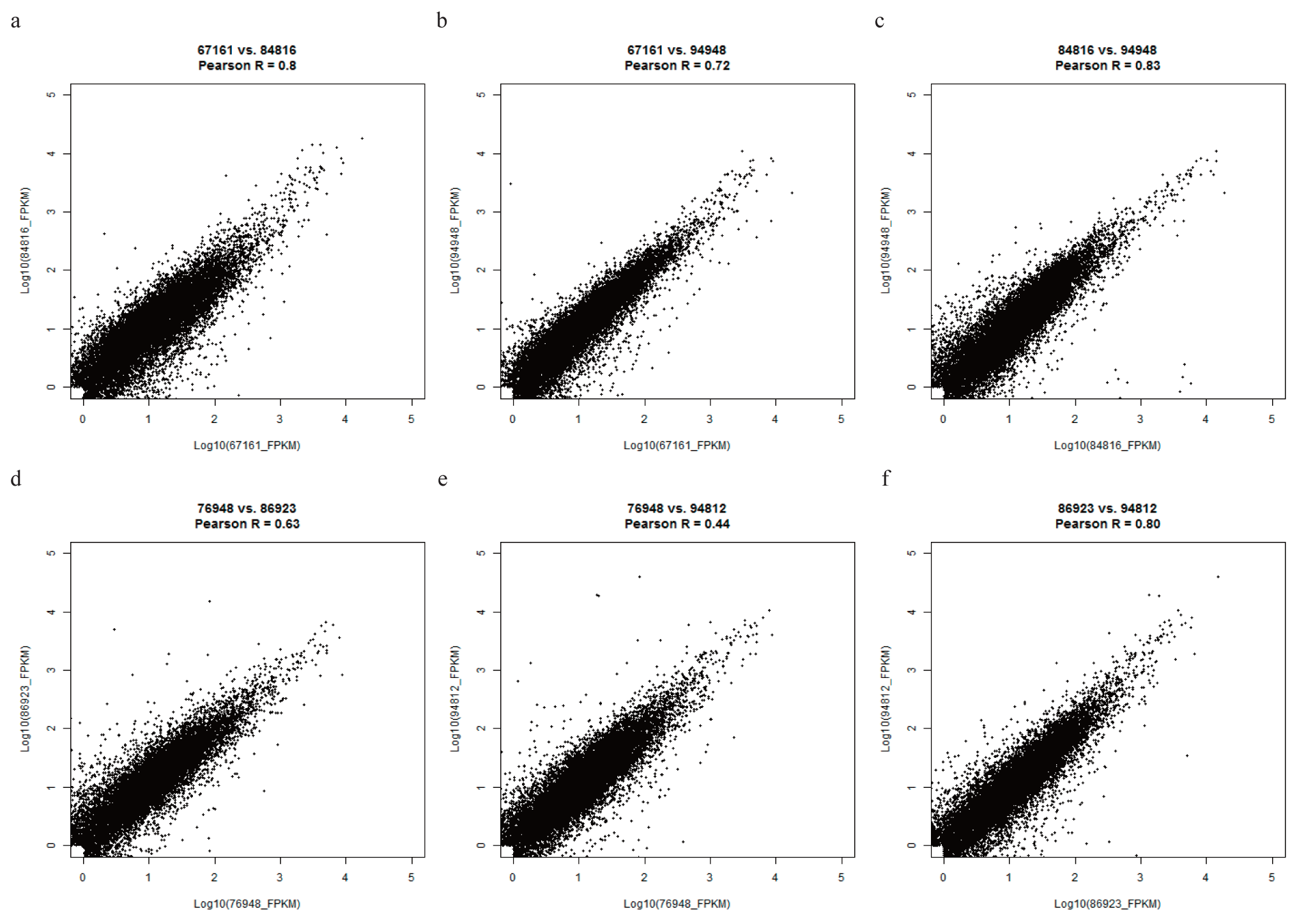

Supplement: Additional file 3: — Scatter plots of gene expression values between samples. A figure illustrating how similar of genes expressed among 6 samples. Panels a, b, and c show comparisons among 67161, 94948, and 84816 from the NSLN negative group. These three samples display very similar gene expression levels based on Pearson correlations. Panels d, e, and f show comparisons among 76948, 86923, and 94812 from the NSLN positive group. Gene expression levels in 76948 are quite dissimilar compared with those in 86923 and 94812 based on their Pearson correlations. [file 12957_2015_642_MOESM3_ESM.tif]

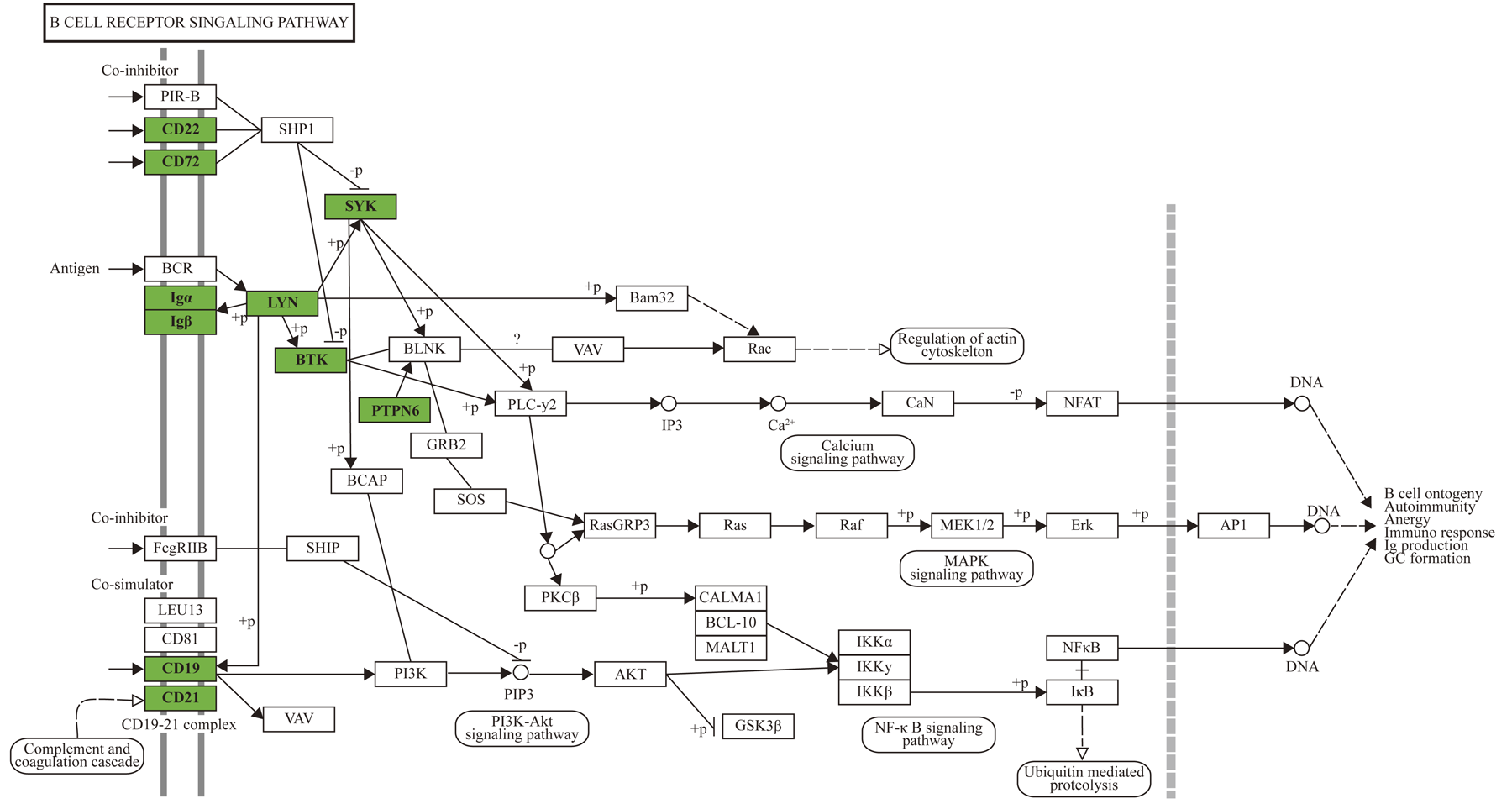

Supplement: Additional file 7: — A KEGG pathway figure for the enriched pathway B cell receptor signaling pathway in down-regulated genes. A figure illustrating the pathway of down-regulated genes enriched. The genes colored with green were down-regulated in NSLN positive group. [file 12957_2015_642_MOESM7_ESM.tif]

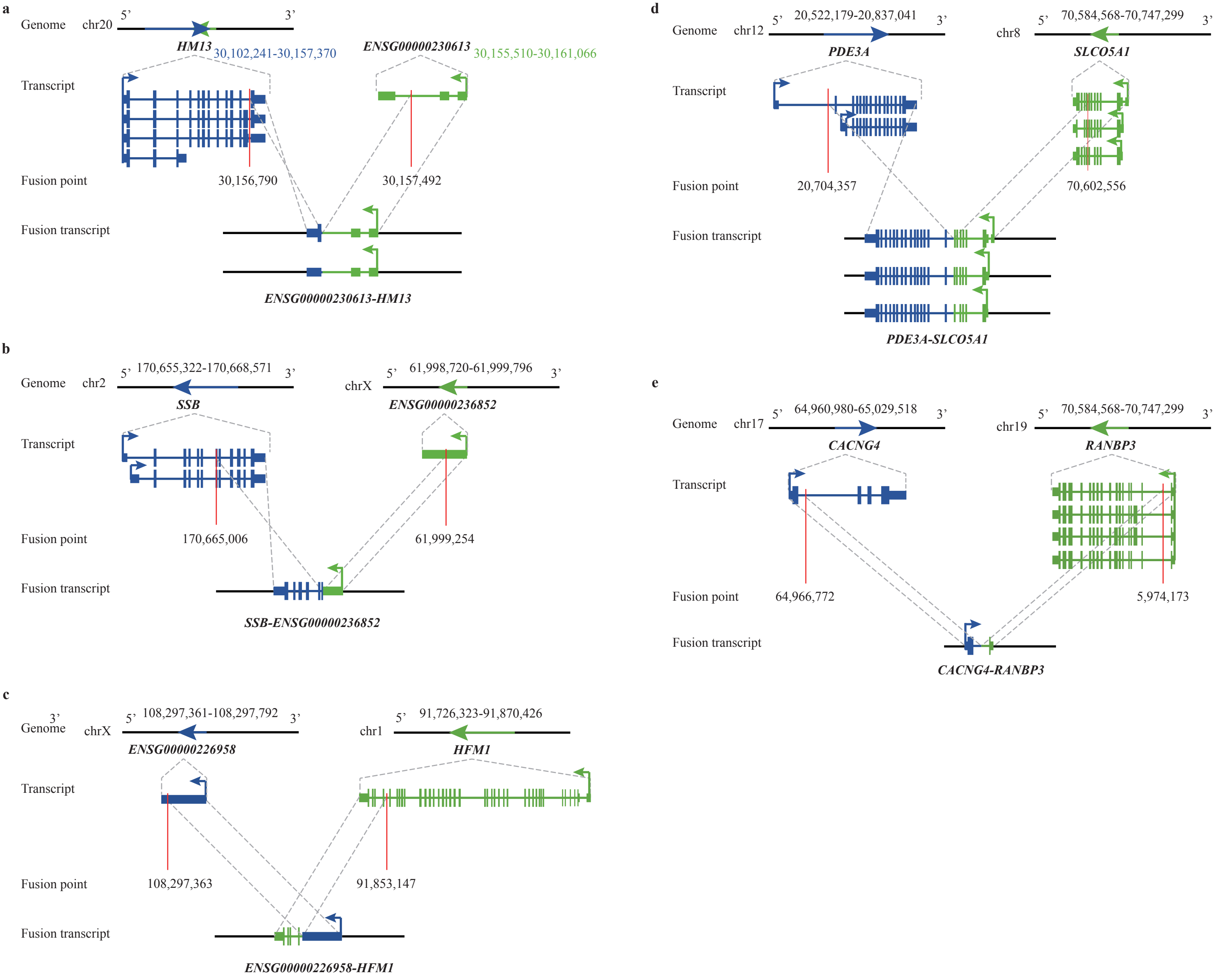

Supplement: Additional file 8: — The schematic diagram of other fused genes. A figure showing how the rest of gene fusions occurred. The two genes that fused together were shown in blue and green. There fused point was shown in a vertical red line. [file 12957_2015_642_MOESM8_ESM.pdf]
