# Supplementary material for: Molecular biomarkers screened by next-generation RNA sequencing for non-sentinel lymph node status prediction in breast cancer patients with metastatic sentinel lymph nodes
Source: World J Surg Oncol. 2015 Aug 28;13:258. doi: 10.1186/s12957-015-0642-2 (PMC4551378; doi:10.1186/s12957-015-0642-2)
Supplement: Additional file 4: — Classification of genes based on the expression levels. A table showing how the gene expression levels were classified. [file 12957_2015_642_MOESM4_ESM.doc]

Additional file 4 Classification of genes based on the expression levels

| Classification | Decile | FPKM (NSLN negative) | FPKM (NSLN positive) |
| --- | --- | --- | --- |
| Lowly | Min | 1 | 1 |
| Lowly | 1^st^ | 2.22 | 2.12 |
| Lowly | 2^nd^ | 4.32 | 3.84 |
| Lowly | 3^rd^ | 7.4 | 6.1 |
| Moderately | 4^th^ | 11.5 | 8.83 |
| Moderately | 5^th^ | 1663 | 12.39 |
| Moderately | 6^th^ | 24.44 | 17.53 |
| Moderately | 7^th^ | 36.63 | 25.2 |
| Highly | 8^th^ | 56.72 | 34.48 |
| Highly | 9^th^ | 109.06 | 74.4 |
| Highly | Max | 20627 | 22301 |
