# Supplementary material for: Molecular biomarkers screened by next-generation RNA sequencing for non-sentinel lymph node status prediction in breast cancer patients with metastatic sentinel lymph nodes
Source: World J Surg Oncol. 2015 Aug 28;13:258. doi: 10.1186/s12957-015-0642-2 (PMC4551378; doi:10.1186/s12957-015-0642-2)
Supplement: Additional file 5: — Specifically expressed genes in the NSLN negative and –positive groups. A table showing the FPKM values of specifically expressed genes in two groups. [file 12957_2015_642_MOESM5_ESM.doc]

Additional file 5 Specifically expressed genes in the NSLN negative and –positive groups

| Gene | FPKM_NSLN-negative | FPKM_NLN-positive | Category |
| --- | --- | --- | --- |
| **NSLN-positive-specific** |  |  |  |
| MIR3936 | 0 | 2210.61 | Highly |
| MIR223 | 0 | 7256.4 | Highly |
| SNORA3 | 0 | 569.001 | Highly |
| SNORA18 | 0 | 433.826 | Highly |
| MIR941-3 | 0 | 409.65 | Highly |
| SNORA7B | 0 | 143.191 | Highly |
| SNORA13 | 0 | 131.596 | Highly |
| SNORA2A | 0 | 113.79 | Highly |
| SCARNA11 | 0 | 112.023 | Highly |
| MIR3907 | 0 | 76.0259 | Highly |
| SNORA84 | 0 | 66.7111 | Highly |
| CYP2A13 | 0 | 39.565 | Highly |
| SNORA62 | 0 | 38.9325 | Highly |
| HOXB-AS3 | 0 | 14.3954 | Moderately |
| H3F3A | 0 | 6.73902 | Moderately |
| LOC152225 | 0 | 5.90847 | Lowly |
| KCNJ3 | 0 | 5.29505 | Lowly |
| MT1DP | 0 | 4.69948 | Lowly |
| LINC00305 | 0 | 3.51422 | Lowly |
| CTAG2 | 0 | 2.85083 | Lowly |
| KRT27 | 0 | 2.7179 | Lowly |
| KRTAP3-3 | 0 | 2.68884 | Lowly |
| MMP3 | 0 | 2.26602 | Lowly |
| SERPINA11 | 0 | 2.23633 | Lowly |
| C11orf88 | 0 | 2.10716 | Lowly |
| MCCD1 | 0 | 1.87629 | Lowly |
| HIST1H4F | 0 | 1.83801 | Lowly |
| CFC1 | 0 | 1.71168 | Lowly |
| SNORA58 | 0 | 1.64749 | Lowly |
| WFDC6 | 0 | 1.63248 | Lowly |
| CRISP2 | 0 | 1.53846 | Lowly |
| GPRASP2 | 0 | 1.51279 | Lowly |
| HSP90AA6P | 0 | 1.50894 | Lowly |
| SPINK14 | 0 | 1.50115 | Lowly |
| FAM25C | 0 | 1.49643 | Lowly |
| DMRTC2 | 0 | 1.47846 | Lowly |
| HBE1 | 0 | 1.45234 | Lowly |
| TNP1 | 0 | 1.33773 | Lowly |
| ZNF812 | 0 | 1.26748 | Lowly |
| LOC285103 | 0 | 1.22425 | Lowly |
| XAGE2 | 0 | 1.2178 | Lowly |
| KRT26 | 0 | 1.18909 | Lowly |
| NME1-NME2 | 0 | 1.15777 | Lowly |
| GNRH2 | 0 | 1.14409 | Lowly |
| ACCN1 | 0 | 1.09755 | Lowly |
| FAM83A | 0 | 1.04621 | Lowly |
| CFC1B | 0 | 1.00471 | Lowly |
| **NSLN-negative-specific** |  |  |  |
| SNORD89 | 20306.9 | 0 | Highly |
| MIR499A | 683.395 | 0 | Highly |
| SNORA51 | 452.645 | 0 | Highly |
| SNORA40 | 419.292 | 0 | Highly |
| SNORA46 | 281.121 | 0 | Highly |
| SCARNA3 | 206.144 | 0 | Highly |
| SNORA27 | 181.431 | 0 | Highly |
| SNORA32 | 99.9085 | 0 | Highly |
| FABP1 | 84.153 | 0 | Highly |
| MIR941-2 | 43.3094 | 0 | Highly |
| MIR941-4 | 43.3094 | 0 | Highly |
| SNORA1 | 43.0823 | 0 | Highly |
| SNORA80B | 30.3728 | 0 | Moderately |
| SNORA38 | 29.2664 | 0 | Moderately |
| SNORA20 | 19.9907 | 0 | Moderately |
| SCARNA22 | 17.7144 | 0 | Moderately |
| SCARNA18 | 17.3736 | 0 | Moderately |
| SNORA59A | 16.9284 | 0 | Moderately |
| SNORA59B | 16.8318 | 0 | Moderately |
| SNORA50 | 15.1121 | 0 | Moderately |
| SNORA2B | 14.1705 | 0 | Moderately |
| SNORA34 | 14.1705 | 0 | Moderately |
| DEFB131 | 11.7056 | 0 | Moderately |
| RNU4ATAC | 11.6617 | 0 | Moderately |
| OSTN | 11.3438 | 0 | Moderately |
| SNORA6 | 10.9173 | 0 | Moderately |
| SPINK1 | 9.4117 | 0 | Moderately |
| SNORA22 | 8.64999 | 0 | Moderately |
| GSTA1 | 8.60305 | 0 | Moderately |
| SNORA16A | 8.58544 | 0 | Moderately |
| SNORA60 | 7.53578 | 0 | Moderately |
| SNORA71B | 7.52259 | 0 | Moderately |
| CLCA1 | 7.36275 | 0 | Moderately |
| HTN3 | 7.28569 | 0 | Moderately |
| SNORA5C | 6.93829 | 0 | Moderately |
| LCE3D | 6.69973 | 0 | Moderately |
| GKN1 | 6.61751 | 0 | Moderately |
| HCG23 | 6.61658 | 0 | Moderately |
| DEFB109P1B | 4.96014 | 0 | Moderately |
| PRAP1 | 4.63185 | 0 | Moderately |
| SPRR1B | 4.5961 | 0 | Moderately |
| RPL13AP5 | 4.53595 | 0 | Moderately |
| OR2M4 | 4.46401 | 0 | Moderately |
| TMPRSS15 | 4.1391 | 0 | Moderately |
| C11orf86 | 4.13475 | 0 | Moderately |
| LOC100129216 | 3.86838 | 0 | Moderately |
| ATP6V1G3 | 3.80056 | 0 | Moderately |
| ASCL3 | 3.64433 | 0 | Moderately |
| LINC00483 | 3.63309 | 0 | Moderately |
| SCGB1D4 | 3.59575 | 0 | Moderately |
| ATP6V1G2-DDX39B | 3.48663 | 0 | Moderately |
| DEFA5 | 3.38617 | 0 | Moderately |
| HTN1 | 3.31553 | 0 | Moderately |
| CHP2 | 3.1342 | 0 | Moderately |
| NCOR1P1 | 2.89226 | 0 | Moderately |
| SMR3B | 2.78551 | 0 | Moderately |
| PNLIPRP2 | 2.77411 | 0 | Moderately |
| GAL | 2.75484 | 0 | Moderately |
| SLC28A2 | 2.64934 | 0 | Moderately |
| FGF23 | 2.4059 | 0 | Moderately |
| RPS4Y1 | 2.32998 | 0 | Moderately |
| HIST1H4L | 2.30895 | 0 | Moderately |
| LOC100216001 | 2.23951 | 0 | Moderately |
| IRX4 | 2.23458 | 0 | Moderately |
| SI | 2.14254 | 0 | Moderately |
| LOC100505679 | 2.09321 | 0 | Moderately |
| PDX1 | 1.94707 | 0 | Moderately |
| C10orf82 | 1.89259 | 0 | Moderately |
| SERPINB3 | 1.88256 | 0 | Moderately |
| SPRR1A | 1.86039 | 0 | Moderately |
| MT1JP | 1.83852 | 0 | Moderately |
| C8orf22 | 1.81399 | 0 | Moderately |
| GSTA5 | 1.70466 | 0 | Moderately |
| ESX1 | 1.6991 | 0 | Moderately |
| UGT1A6 | 1.67178 | 0 | Moderately |
| LOC100129515 | 1.66681 | 0 | Moderately |
| NKX2-1 | 1.64122 | 0 | Moderately |
| LINC00052 | 1.64038 | 0 | Moderately |
| GPR128 | 1.57 | 0 | Moderately |
| MOGAT3 | 1.56502 | 0 | Moderately |
| LOC642426 | 1.50417 | 0 | Moderately |
| C10orf91 | 1.48539 | 0 | Moderately |
| RLN3 | 1.48524 | 0 | Moderately |
| H2AFB2 | 1.39714 | 0 | Moderately |
| DEFA6 | 1.38822 | 0 | Moderately |
| GRP | 1.38128 | 0 | Moderately |
| LOC347411 | 1.37977 | 0 | Moderately |
| C4orf6 | 1.34868 | 0 | Moderately |
| CALCB | 1.34627 | 0 | Moderately |
| MIR7-3HG | 1.33644 | 0 | Moderately |
| GK2 | 1.30254 | 0 | Moderately |
| LRRC3B | 1.26447 | 0 | Moderately |
| KRTAP7-1 | 1.24184 | 0 | Moderately |
| SYCN | 1.19928 | 0 | Moderately |
| REXO1L2P | 1.16484 | 0 | Moderately |
| ARMS2 | 1.16229 | 0 | Moderately |
| LALBA | 1.15999 | 0 | Moderately |
| CLC | 1.13623 | 0 | Moderately |
| IGFBP1 | 1.10978 | 0 | Moderately |
| FGG | 1.10136 | 0 | Moderately |
| OR6B2 | 1.09767 | 0 | Moderately |
| CEACAM8 | 1.0303 | 0 | Moderately |
| SPANXB2 | 1.02279 | 0 | Moderately |
