# Supplementary material for: Molecular biomarkers screened by next-generation RNA sequencing for non-sentinel lymph node status prediction in breast cancer patients with metastatic sentinel lymph nodes
Source: World J Surg Oncol. 2015 Aug 28;13:258. doi: 10.1186/s12957-015-0642-2 (PMC4551378; doi:10.1186/s12957-015-0642-2)
Supplement: Additional file 6: — Expression levels of 98 down-regulated genes and 62 up-regulated genes in the NSLN positive group. A table showing the expression values of regulated genes. [file 12957_2015_642_MOESM6_ESM.doc]

Additional file 6 Expression levels of 98 down-regulated genes and 62 up-regulated genes in the NSLN positive group

| Gene | Regulation^a^ | NSLN  Negative | NSLN  Positive | Log2(FC)^b^ | P_value | FDR |
| --- | --- | --- | --- | --- | --- | --- |
| KRT20 | Down | 17.185 | 0.070 | -7.948 | 3.78E-07 | 0.000193 |
| KRT4 | Down | 6.421 | 0.046 | -7.139 | 0.000591 | 0.047805 |
| VPREB1 | Down | 25.933 | 0.189 | -7.097 | 7.89E-05 | 0.011731 |
| RBP2 | Down | 31.243 | 0.579 | -5.753 | 4.37E-06 | 0.001364 |
| ALDOB | Down | 4.638 | 0.092 | -5.657 | 7.56E-05 | 0.011482 |
| BIRC7 | Down | 7.801 | 0.164 | -5.570 | 0.000141 | 0.016953 |
| FIBCD1 | Down | 7.746 | 0.186 | -5.379 | 2.27E-06 | 0.000906 |
| MUC13 | Down | 8.845 | 0.234 | -5.239 | 8.23E-06 | 0.002191 |
| FCAMR | Down | 50.504 | 1.381 | -5.193 | 1.55E-15 | 4.97E-12 |
| DHRS2 | Down | 19.044 | 0.554 | -5.103 | 4.81E-05 | 0.008534 |
| OR7D2 | Down | 3.608 | 0.106 | -5.088 | 0.000117 | 0.015371 |
| GBA3 | Down | 4.859 | 0.145 | -5.070 | 0.000272 | 0.028304 |
| TM4SF4 | Down | 12.618 | 0.429 | -4.880 | 7.53E-05 | 0.011482 |
| PVALB | Down | 56.824 | 2.053 | -4.791 | 3.36E-05 | 0.006705 |
| KCNG1 | Down | 6.123 | 0.224 | -4.771 | 0.000152 | 0.017924 |
| SIGLEC14 | Down | 25.268 | 0.926 | -4.770 | 1.15E-05 | 0.002878 |
| REG4 | Down | 17.462 | 0.768 | -4.507 | 8.62E-05 | 0.012665 |
| FCER2 | Down | 381.639 | 19.725 | -4.274 | 3.52E-08 | 3.21E-05 |
| SERPINA9 | Down | 18.063 | 0.958 | -4.237 | 1.10E-07 | 7.42E-05 |
| RHBG | Down | 6.874 | 0.373 | -4.205 | 0.000589 | 0.047805 |
| PAX5 | Down | 30.656 | 1.705 | -4.168 | 3.71E-06 | 0.001217 |
| TNFRSF13C | Down | 102.065 | 5.922 | -4.107 | 7.80E-06 | 0.002168 |
| HTR3A | Down | 11.087 | 0.689 | -4.008 | 0.000378 | 0.03552 |
| FBN3 | Down | 1.975 | 0.124 | -3.994 | 0.000398 | 0.036112 |
| SHISA8 | Down | 60.411 | 4.218 | -3.840 | 4.07E-05 | 0.007504 |
| INSM1 | Down | 7.404 | 0.560 | -3.724 | 0.000275 | 0.028304 |
| FAM129C | Down | 281.760 | 21.727 | -3.697 | 2.54E-06 | 0.000954 |
| MYO7B | Down | 4.234 | 0.331 | -3.679 | 0.000133 | 0.016376 |
| HBA2 | Down | 261.165 | 20.388 | -3.679 | 0.000352 | 0.034094 |
| NOG | Down | 20.450 | 1.710 | -3.580 | 0.000208 | 0.02313 |
| KRT72 | Down | 85.779 | 7.566 | -3.503 | 9.49E-05 | 0.013475 |
| LOC283663 | Down | 120.222 | 11.193 | -3.425 | 2.95E-05 | 0.006085 |
| CD72 | Down | 135.460 | 12.669 | -3.419 | 2.49E-05 | 0.005299 |
| CD19 | Down | 230.117 | 21.704 | -3.406 | 3.06E-06 | 0.001104 |
| NCF1C | Down | 175.794 | 17.802 | -3.304 | 0.000144 | 0.017155 |
| LYL1 | Down | 183.268 | 20.131 | -3.186 | 5.29E-05 | 0.009008 |
| CD22 | Down | 405.021 | 46.091 | -3.135 | 6.45E-07 | 0.000294 |
| LY9 | Down | 183.292 | 21.502 | -3.092 | 4.31E-06 | 0.001364 |
| FGFR4 | Down | 30.500 | 3.652 | -3.062 | 5.57E-05 | 0.009008 |
| CEACAM21 | Down | 17.885 | 2.148 | -3.058 | 0.000125 | 0.015987 |
| LINC00494 | Down | 32.417 | 4.007 | -3.016 | 0.000395 | 0.036058 |
| ALPK2 | Down | 11.103 | 1.414 | -2.973 | 0.000434 | 0.038552 |
| ACCN3 | Down | 26.618 | 3.597 | -2.887 | 6.95E-08 | 5.23E-05 |
| LOC606724 | Down | 790.178 | 107.475 | -2.878 | 9.30E-06 | 0.002411 |
| VPREB3 | Down | 445.649 | 62.041 | -2.845 | 0.000315 | 0.031492 |
| FCRLA | Down | 145.881 | 20.351 | -2.842 | 6.84E-06 | 0.001988 |
| SEMA7A | Down | 54.734 | 7.811 | -2.809 | 0.000223 | 0.024151 |
| PARP15 | Down | 99.133 | 14.400 | -2.783 | 0.000295 | 0.029714 |
| CREM | Down | 75.876 | 11.783 | -2.687 | 0.000384 | 0.035825 |
| BLK | Down | 135.049 | 21.273 | -2.666 | 0.000366 | 0.034936 |
| FDCSP | Down | 811.835 | 129.809 | -2.645 | 0.000595 | 0.047834 |
| BCL11A | Down | 37.068 | 5.943 | -2.641 | 0.00014 | 0.016953 |
| CXorf65 | Down | 83.627 | 14.230 | -2.555 | 1.05E-08 | 1.03E-05 |
| ACVR1C | Down | 2.030 | 0.349 | -2.541 | 2.56E-05 | 0.005374 |
| SIGLEC10 | Down | 60.853 | 10.584 | -2.523 | 0.000357 | 0.034265 |
| ABCB4 | Down | 4.647 | 0.813 | -2.516 | 4.78E-06 | 0.001456 |
| BCL2A1 | Down | 109.119 | 19.183 | -2.508 | 1.88E-07 | 0.000114 |
| MAGED4B | Down | 21.659 | 3.924 | -2.465 | 0.000241 | 0.025927 |
| IL9R | Down | 10.869 | 1.981 | -2.456 | 3.09E-05 | 0.006271 |
| SP140 | Down | 64.742 | 11.919 | -2.441 | 0.000123 | 0.015987 |
| DOK3 | Down | 172.113 | 31.709 | -2.440 | 0.000258 | 0.027407 |
| CDH23 | Down | 37.036 | 7.144 | -2.374 | 4.30E-07 | 0.000212 |
| PRKCB | Down | 68.034 | 13.309 | -2.354 | 2.99E-09 | 3.48E-06 |
| MBNL3 | Down | 8.219 | 1.662 | -2.306 | 0.000583 | 0.047802 |
| GLYCTK | Down | 26.183 | 5.451 | -2.264 | 8.04E-06 | 0.002187 |
| IKZF3 | Down | 17.926 | 3.928 | -2.190 | 2.34E-11 | 4.98E-08 |
| GRIN1 | Down | 3.111 | 0.714 | -2.124 | 5.23E-08 | 4.46E-05 |
| SIRPG | Down | 115.231 | 27.636 | -2.060 | 0.000287 | 0.029371 |
| RIC3 | Down | 10.733 | 2.641 | -2.023 | 6.59E-05 | 0.010395 |
| LMF1 | Down | 130.838 | 32.239 | -2.021 | 0.000471 | 0.040364 |
| ZNF331 | Down | 38.798 | 9.654 | -2.007 | 2.95E-07 | 0.000157 |
| TEKT4P2 | Down | 207.592 | 52.661 | -1.979 | 1.63E-06 | 0.000696 |
| NFATC1 | Down | 43.500 | 11.282 | -1.947 | 0.000133 | 0.016376 |
| LAT | Down | 398.759 | 103.749 | -1.942 | 0.000394 | 0.036058 |
| PLCXD1 | Down | 71.197 | 18.761 | -1.924 | 4.34E-05 | 0.007822 |
| ANK1 | Down | 3.845 | 1.029 | -1.902 | 1.98E-10 | 3.16E-07 |
| ZBTB20 | Down | 3.878 | 1.077 | -1.849 | 2.22E-16 | 9.46E-13 |
| ACHE | Down | 24.757 | 7.410 | -1.740 | 7.55E-06 | 0.002145 |
| C1QTNF6 | Down | 48.491 | 15.281 | -1.666 | 6.51E-11 | 1.19E-07 |
| FLT3LG | Down | 187.225 | 59.698 | -1.649 | 0.00027 | 0.028264 |
| KIAA1908 | Down | 6.338 | 2.117 | -1.582 | 3.34E-06 | 0.001154 |
| IRF5 | Down | 53.493 | 18.109 | -1.563 | 4.75E-10 | 6.74E-07 |
| RELL2 | Down | 15.792 | 5.349 | -1.562 | 0.000177 | 0.019985 |
| TDRD10 | Down | 12.450 | 4.225 | -1.559 | 1.91E-12 | 4.88E-09 |
| ALKBH6 | Down | 105.413 | 36.601 | -1.526 | 0.000515 | 0.042729 |
| NR3C1 | Down | 33.160 | 11.994 | -1.467 | 0.000111 | 0.014897 |
| ANKRD6 | Down | 8.493 | 3.140 | -1.436 | 6.09E-06 | 0.001811 |
| PDLIM2 | Down | 87.748 | 32.457 | -1.435 | 2.05E-07 | 0.000119 |
| MASTL | Down | 7.993 | 2.964 | -1.431 | 2.45E-06 | 0.000947 |
| PYHIN1 | Down | 10.954 | 4.174 | -1.392 | 0.000344 | 0.033656 |
| C15orf41 | Down | 6.758 | 2.684 | -1.332 | 0.00044 | 0.038552 |
| TBC1D1 | Down | 39.262 | 15.669 | -1.325 | 0 | 0 |
| ZNF767 | Down | 48.225 | 19.264 | -1.324 | 0 | 0 |
| CEP78 | Down | 7.762 | 3.119 | -1.315 | 9.36E-05 | 0.013449 |
| DISC1 | Down | 4.679 | 1.956 | -1.258 | 9.43E-06 | 0.002411 |
| CSTF3 | Down | 88.651 | 38.179 | -1.215 | 0.000345 | 0.033656 |
| CD101 | Down | 3.635 | 1.680 | -1.113 | 5.20E-05 | 0.00899 |
| PTBP3 | Down | 34.730 | 20.118 | -0.788 | 0.000109 | 0.01486 |
| KLK11 | up | 0.929 | 185.484 | 7.641 | 2.66E-07 | 0.000148 |
| SCGB3A1 | up | 8.062 | 1231.390 | 7.255 | 6.12E-08 | 4.89E-05 |
| CLEC3A | up | 0.984 | 143.594 | 7.190 | 2.31E-09 | 2.96E-06 |
| CYP2A6 | up | 0.454 | 63.311 | 7.124 | 6.09E-07 | 0.000288 |
| KLK10 | up | 0.171 | 19.113 | 6.803 | 1.46E-05 | 0.003394 |
| KLK12 | up | 0.892 | 94.900 | 6.733 | 3.11E-06 | 0.001104 |
| KLK13 | up | 0.572 | 49.975 | 6.449 | 1.10E-07 | 7.42E-05 |
| CYP2A7 | up | 0.330 | 26.368 | 6.321 | 2.34E-05 | 0.005158 |
| OBP2B | up | 0.427 | 33.387 | 6.289 | 0.000453 | 0.039105 |
| KCNC2 | up | 0.318 | 21.596 | 6.085 | 1.77E-05 | 0.004046 |
| CPB1 | up | 1.688 | 106.871 | 5.984 | 1.23E-05 | 0.002955 |
| CHGB | up | 0.782 | 48.768 | 5.963 | 3.49E-06 | 0.001175 |
| C8orf85 | up | 3.825 | 194.220 | 5.666 | 1.20E-05 | 0.002955 |
| TAT | up | 0.241 | 10.284 | 5.416 | 0.000259 | 0.027407 |
| SERPINA3 | up | 8.764 | 347.481 | 5.309 | 1.95E-06 | 0.000805 |
| NR0B1 | up | 0.466 | 17.811 | 5.256 | 0.000194 | 0.021807 |
| NXPH1 | up | 0.119 | 4.056 | 5.088 | 0.000538 | 0.044361 |
| USH1C | up | 0.152 | 5.004 | 5.042 | 0.000479 | 0.040531 |
| BPIFB2 | up | 0.599 | 19.406 | 5.017 | 4.11E-05 | 0.007504 |
| BMPR1B | up | 2.962 | 91.718 | 4.953 | 1.63E-06 | 0.000696 |
| GRIA2 | up | 0.201 | 5.580 | 4.797 | 9.17E-05 | 0.013327 |
| PIP | up | 210.657 | 4999.240 | 4.569 | 0.000124 | 0.015987 |
| CLIC6 | up | 0.966 | 21.990 | 4.509 | 6.96E-05 | 0.010854 |
| KRT23 | up | 0.687 | 15.606 | 4.506 | 0.000216 | 0.023846 |
| PPP1R3C | up | 2.042 | 42.455 | 4.378 | 0.000109 | 0.01486 |
| MUCL1 | up | 32.118 | 632.156 | 4.299 | 0.000432 | 0.038552 |
| KRT15 | up | 10.619 | 204.872 | 4.270 | 0.000419 | 0.037752 |
| CALML5 | up | 10.770 | 206.186 | 4.259 | 3.98E-05 | 0.00749 |
| CHAD | up | 7.624 | 134.646 | 4.143 | 3.72E-05 | 0.007198 |
| DNAH5 | up | 0.113 | 1.951 | 4.115 | 0.000163 | 0.018587 |
| ABCC13 | up | 1.021 | 17.599 | 4.108 | 0.000345 | 0.033656 |
| ANKRD30A | up | 1.393 | 24.018 | 4.108 | 1.39E-05 | 0.003292 |
| CA8 | up | 0.945 | 15.425 | 4.029 | 0.00044 | 0.038552 |
| ANKRD30B | up | 0.542 | 8.491 | 3.970 | 0.000156 | 0.018164 |
| TDRD5 | up | 1.196 | 17.486 | 3.870 | 5.54E-05 | 0.009008 |
| TFPI2 | up | 8.578 | 124.148 | 3.855 | 5.04E-05 | 0.008833 |
| PROM1 | up | 0.409 | 5.831 | 3.833 | 0.000223 | 0.024151 |
| GPRC5A | up | 2.196 | 29.088 | 3.728 | 0.000107 | 0.01486 |
| IGFBP2 | up | 17.395 | 228.681 | 3.717 | 5.40E-05 | 0.009008 |
| SHROOM1 | up | 6.236 | 81.906 | 3.715 | 8.51E-09 | 9.07E-06 |
| RBM24 | up | 1.737 | 21.524 | 3.631 | 1.83E-05 | 0.004106 |
| S100P | up | 21.321 | 254.486 | 3.577 | 0.000487 | 0.040934 |
| C15orf48 | up | 16.410 | 194.661 | 3.568 | 7.64E-05 | 0.011482 |
| IGSF21 | up | 1.504 | 17.788 | 3.564 | 0.000474 | 0.040364 |
| STEAP2 | up | 0.743 | 8.452 | 3.509 | 2.41E-05 | 0.005228 |
| PARD6B | up | 6.484 | 61.761 | 3.252 | 6.28E-05 | 0.010031 |
| CXADRP3 | up | 6.090 | 54.482 | 3.161 | 0.00045 | 0.039098 |
| ZG16B | up | 24.991 | 201.560 | 3.012 | 0.000159 | 0.018309 |
| SHROOM3 | up | 1.011 | 7.950 | 2.975 | 0.00029 | 0.029399 |
| FOXA1 | up | 13.389 | 101.173 | 2.918 | 0.000128 | 0.016208 |
| GREB1 | up | 2.207 | 15.950 | 2.853 | 0.000103 | 0.014484 |
| AGTR1 | up | 3.884 | 27.816 | 2.840 | 0.000112 | 0.014897 |
| DEGS2 | up | 12.305 | 87.073 | 2.823 | 0.000373 | 0.035365 |
| SCNN1A | up | 8.068 | 55.712 | 2.788 | 0.000132 | 0.016376 |
| PFN2 | up | 12.336 | 81.482 | 2.724 | 0.000387 | 0.03589 |
| SYBU | up | 5.102 | 31.039 | 2.605 | 5.46E-05 | 0.009008 |
| FXYD3 | up | 58.123 | 351.381 | 2.596 | 0.000496 | 0.041474 |
| AR | up | 2.299 | 10.617 | 2.207 | 0.000606 | 0.048396 |
| NEBL | up | 2.698 | 9.990 | 1.888 | 3.83E-05 | 0.007313 |
| PROM2 | up | 6.818 | 24.265 | 1.832 | 3.60E-05 | 0.007072 |
| MUC1 | up | 511.455 | 1766.890 | 1.789 | 1.64E-07 | 0.000105 |
| LOC440335 | up | 72.017 | 242.722 | 1.753 | 0.000153 | 0.017924 |

Note: ^a^ represents that the relative expression levels (up/down) in NSLN positive samples against NSLN negative samples. ^b^ FC=fold change of (NLSN positive/negative)
